# Health-related quality of life in refugee youth and the mediating role of mental distress and post-migration stressors

**DOI:** 10.1007/s11136-021-02811-7

**Published:** 2021-03-13

**Authors:** Cecilie Dangmann, Øivind Solberg, Per Normann Andersen

**Affiliations:** 1 Faculty of Health and Social Sciences, Inland Norway University of Applied Sciences, Hamarvegen 112, 2418 Elverum, NO Norway; 2 Section for Implementation and Treatment Research, Norwegian Centre for Violence and Traumatic Stress Studies, Oslo, Norway; 3 Department of Psychology, Inland Norway University of Applied Sciences, Lillehammer, Norway

**Keywords:** Health-related quality of life, Mental distress, Post-migration stressors, Post-traumatic stress disorder, Refugee, Youth

## Abstract

**Purpose:**

The aim of this study is to explore how potentially traumatic events (PTEs) from war and flight influence health-related quality of life (HRQoL) in young refugees after recent resettlement. In a model based on earlier theory, we tested if post-migration stressors and mental distress mediated the effect of PTEs on HRQoL, individually and in serial. We also explored how different types of post-migration stressors influenced different dimensions of HRQoL.

**Methods:**

This study used a cross-sectional design where 160 Syrian youth recently resettled in Norway completed questionnaires at school between May and December 2018. Correlations between types of post-migration stressors and dimensions of HRQoL were explored and a serial multiple mediator model was tested. Models were adjusted for age and gender, using two types of mental distress; post-traumatic stress disorder (PTSD) and general mental distress.

**Results:**

Higher levels of PTEs reduced experienced HRQoL, but this direct effect was mediated by post-migration stressors alone and in serial with mental distress. Despite high levels of mental distress, this did not affect HRQoL independently, only in serial mediation with increased post-migration stressors. Economic concerns and discrimination were types of post-migration stressors affecting several dimensions of HRQoL.

**Conclusion:**

Quality of life in refugee is affected by past events from war, stressors in current resettlement and elevated mental distress through complex interrelations. The study reiterates the importance of considering structural and everyday post-migration stressors in policy and intervention to improve the health and wellbeing of refugee youth.

## Introduction

Forced migration due to conflict is increasing worldwide, resulting in people seeking refuge in other countries. The war in Syria has led to one of the largest mass migrations since the WWII with no signs of abating, and an estimated half of these are children and youth [1]. It is well documented that refugee children and youth are vulnerable to mental distress such as depression, anxiety and post-traumatic stress disorder (PTSD). This mental distress has been related to experiences of potentially traumatic events (PTEs) during war and flight, such as war action, witnessing death and violence [2, 3]. Similar results are described in studies of children and youth from Syria where the majority have experienced several PTEs [4–6]. Additionally, findings suggest that the impact of war and forced migration on mental health is compounded or alleviated by the post-migration resettlement context^1^ [3]. A constellation of stressors related to resettlement in a new country, such as discrimination, uncertainties related to asylum status, poor economy, lack of social support, parental illness and acculturation, have shown both a cumulative effect and differential associations depending on type of stressor [7–9]. These stressors have shown significance above and beyond pre-migration PTEs, even more prominently so in children and youth [7, 8]. Suggesting that previous research overlooked the importance of post-migration stressors, Miller and Rasmussen [10] proposed a model including both previous war exposure and current resettlement stressors to explain mental distress in refugees. This “ecological model of refugee distress” builds on social ecological models such as Bronfenbrenner [11], in which factors at multiple levels influence human development. This model has shown greater explanatory power, however, it is still important to explore the interrelated pathways of these pre- and post-migration factors to understand how and when to implement interventions [3].

Building on earlier stress theories, Miller and Rasmussen [12] argued that the chronicity of daily stressors has the potential to deplete coping mechanisms, affecting the capacity to cope with PTEs. Hence, post-migration stressors seem to have a direct effect on mental distress, as well as indirect effects by preventing recovery and^1^ exacerbating symptoms from trauma [13, 14]. The hypothesis of post-migration stressors mediating, or partially explaining the effect of PTEs on mental distress in refugees, has been supported in several studies [7, 10, 15–18].

**Fig. 1:**
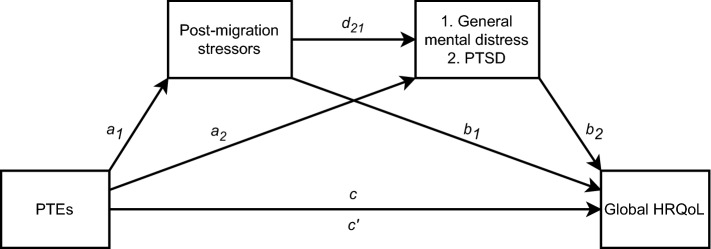
Conceptual serial mediation model. Path c is the total effect of the predictor (Potentially Traumatic Events) and outcome variable (Health-Related Quality of Life) both directly and indirectly through the mediators. Paths a_1_ and a_2_ represent the relationship between the predictors and the mediators (Post-migration stressors and general mental distress/PTSD). Path d_21_ is the relationship between the two mediators. Paths b_1_ and b_2_ indicate the association between the mediators and outcome whilst the predictor value is controlled. The c’ path is the direct effect between the predictor and the outcome excluding the mediator variables. The indirect effect (c–c’ = a_1_b_1_ + a_2_b_2_ + a_1__21_b_2_) is indicated by a statistically significant difference between c and c’. The indirect effect would be significant with CIs not including zero ([30])

However, it is important to note that most refugees do not develop mental health problems [19], and previous research stresses the importance of assessing signs of adaptive functioning or resilience in this group [20]. Concepts suitable for capturing both positive and negative psychological adjustments in this population such as quality of life are therefore important to assess [18, 21]. Quality of life is the individuals’ perceptions of many life dimensions such as their health, relationships, learning and participation [22]. Exploring outcomes relevant to a wider refugee population, not only those suffering from distress, is important within a public health perspective and for services supporting refugees. Yet, it is less investigated in refugee populations, and the results vary. For example, some studies report similar or better quality of life [18, 23, 24] for refugee youth resettled in high-income countries compared to other groups; others find slightly reduced levels [25]. A UN report of Syrian youth in Jordan found greatly reduced levels [26]. Context and measurements could be contributors to these differences, but pre- and post-migration variables are also consistently associated with quality of life [7, 27]. It might therefore be relevant to consider quality of life as an alternative outcome to mental distress, in the “ecological model of refugee distress”. However, the association between mental distress and quality of life has also been repeatedly verified among youth in general [28] and in refugees [27, 29], and purely replacing quality of life as an outcome, would not account for the potential influence of mental distress on quality of life. Hence, this study aims to further explore the “ecological model of refugee distress” by including quality of life as an outcome, and mental distress as an additional mediator (see Fig. 1).

Our first hypothesis was that PTEs negatively influence quality of life, in this study measured as health-related quality of life (HRQoL), the health aspect of quality of life [22]. Our second hypothesis was that post-migration stressors, mediate the effect of PTEs on HRQoL, similar to the “ecological model of refugee distress”. Thirdly, we hypothesised that two types of mental distress (general mental distress and post-traumatic stress disorder (PTSD)) also mediate the effect independent of post-migration stressors. Lastly, assuming there is a relationship between them, we hypothesised that mental distress (both general mental distress and PTSD) and post-migration stressors both act as mediators in a sequence. The order of the mediators will be based on the “ecological model of refugee stress” where mental distress is affected by post-migration stressors, but also reversed as the ordering is unknown. Considering the proposed importance of post-migration stressors in previous research, we also want to explore how different post-migration stressors influence different quality-of-life dimensions.

## Methods

### Participants

Syrians were the largest group of forced migrants to enter Norway at the time of the study, and 5553 were registered in the age groups recruited in 2018 [25]. The inclusion criterion for age was 12–24 years, as they have the right to attend secondary and upper secondary schools. Using strategic sampling to recruit recently resettled youth, 40 schools with introductory classes for newly arrived immigrants were contacted. 23 schools located in nine different regions of Norway agreed to participate. Reasons for not participating were *no response to request* (nine schools), *no Syrian students* (six schools) or *already participating in other studies* (two schools). The participating schools had between one and 23 Syrian students attending. Three students declined to participate due to exam preparations or language difficulties. The recruitment period lasted from May to December 2018 and a total of 160 youth from Syria were included in the final study sample.

### Design and setting

The present study utilised a cross-sectional, questionnaire-based design. Questionnaires were administered in both Arabic and Norwegian. The teachers distributed written information about the study in Arabic and Norwegian to the students in advance, and consent forms to parents with children under the age of 16. The youth consenting to participate completed the questionnaire whilst at school with a researcher present to answer questions.

### Measures

All measures had validated Arabic language versions available and permission to use these were sought from all copyright holders. Measures for PTE and Post-migration stressors were translated from Swedish to Norwegian and reviewed to detect and remove any discrepancies in meaning. A cultural expert reviewed the questionnaire for cultural appropriateness and comprehensibility, and a pilot was conducted in a refugee learning centre.

#### Potentially traumatic events (PTEs)

The Refugee Trauma History Checklist was modified to fit the age of respondents and the context of recent resettlement [31]. The list consisted of 10 dichotomous items (yes/no): witnessing war, being forced to leave friends/family, someone you love disappearing, someone trying to hurt you or someone you love, having a life-threatening illness or injury, lacking food or shelter, having to hide, torture, seeing someone die and other frightening experience where you thought your life was in danger. All positive responses were added as a cumulative score (range 0–10) with higher scores indicating higher number of events experienced.

#### Post-migration stressors

The Post-migratory Stress scale was modified to fit the age of the respondents and the Norwegian context [32]. Ten indicators representing different types of stressors experienced since their arrival in Norway were used: perceived discrimination, language difficulties, economic strain, missing family, family cultural conflicts, feeling lonely, missing previous life, feeling unsafe, worrying about having to move or worrying about having to leave Norway. All indicators were scored on a 5-point Likert scale ranging from 0 (Never) to 4 (Very often) and were added as a cumulative score. Higher scores indicate higher frequencies of experienced stressors, range 0–40, and the Cronbach's alpha was 0.77 in this study.

#### General mental distress

The Hopkins Symptom Checklist (HSCL-10) consists of four items related to anxiety and six related to depression that collectively indicate general mental distress [33]. All items have four response categories ranging from 1 (Not at all) to 4 (Extremely) regarding how much the symptoms bothered the respondents in the past 7 days. The response values are added and then divided by the number of items (range 1–4), higher scores indicate greater symptom load. A cut-off score ≥ 1.85 was used as an indication of general mental distress [33]. Lower thresholds have been suggested for youth, however, the more conservative cut-off was chosen as the average age of our participants were older than the reference group in this study [34]. The HSCL-10 has previously shown satisfactory validity and reliability as a measure of mental distress both in young and displaced populations [35]. The Cronbach's alpha in this study was 0.89.

#### Post-traumatic stress disorder (PTSD)

The Child Revised Impact of Events Scale (CRIES-8) is a screening tool measuring the severity of post-trauma intrusion and avoidance symptoms during the last week. Eight items are rated on a 4-point scale ranging from 0 (not at all), 1, 3 to 5 (often) and then added (range 0–40), with higher scores indicating greater symptom loads. The scale is recommended by the Children of War foundation and cross-culturally validated with good psychometric properties, with a cut-off value of ≥ 17 indicating possible PTSD [36]. The Cronbach's alpha in this study was 0.86.

#### Health-related quality of life (HRQoL)

HRQoL is a multidimensional construct considered to be the health aspect of quality of life that focuses on people’s daily functioning and ability to experience a fulfilling life [22]. KIDSCREEN-27 is a generic self-report measure used to assess subjective HRQoL and is cross-culturally validated in 38 languages [37]. The items are rated on a scale from 1 to 5 for experiences in the last week, grouped into five dimensions: Physical wellbeing (Physical, 5 items), Psychological wellbeing (Psychological, 7 items), Autonomy and Parent relations (Autonomy/Parents, 7 items), Social support and peers (Friends, 4 items), and School environment (School, 4 items). We also used KIDSCREEN-10 as a measure of global HRQoL consisting of 10 of the 27 items from all dimensions. This was previously developed through Rasch analysis ensuring that only items which represent a global, unidimensional latent trait are included [38]. Permission was sought from the KIDSCREEN organisation, and the Arabic and Norwegian versions were downloaded from their member webpages. A scoring algorithm was used to calculate T-scores with a mean of 50 and a standard deviation of 10 with higher scores indicating higher self-rated HRQoL [38]. In this study, Cronbach’s alpha was between 0.82 and 0.88 for all dimensions and 0.82 for KIDSCREEN-10.

### Statistical analyses

Our conceptual model was based on previous theory and included pre-migration PTEs as the main predictor, post-migration stressors and mental distress (1. general mental distress and 2. PTSD) as potential mediators and HRQoL as the outcome (see Fig. 1).

The mediating effect was examined by a regression-based approach in the SPSS PROCESS 3.4 macro (model 6), with 5000 bootstrap samples in the procedure suggested by Hayes [30]. This serial mediation procedure enables the isolation of each mediator’s indirect effect as well as the indirect effect passing through both mediators in a series. Considering that the mediators could act in reverse order to the conceptual model, this was tested in separate models. An indirect effect is assumed to be significant at an alpha level of 0.05 if its 95% confidence interval (CI) does not include zero. Gender and age were controlled for as covariates. Initially, residence time was also controlled for, but as this was not significant in the final models, it was removed to increase power of the estimates. Missing values from KIDSCREEN were replaced according to the KIDSCREEN manual using calculated estimates based on Rasch analyses [38], whilst responses missing two or less items in HSCL-10 or CRIES-8 were replaced with individual means. Responses missing more items were categorised as below cut-off values (CRIES-8 and HSCL-10) when assessing the prevalence as not to inflate frequency of cases but were not included in further analyses. A total of 19 participants opted for a shortened questionnaire due to language difficulties. This shortened questionnaire did not contain HSCL-10, CRIES-8 or post-migration stressors. General descriptive statistics, t-tests and correlation analyses were used to explore the variables.

## Results

### Sample characteristics.

The sample included 160 youth from Syria (37.5% female, mean age 18 years) (Table 1). All participants attended school full time and were located in either local secondary or upper secondary schools or adult learning centres. The majority were living with their parents (75%) and of the ones that were not, mostly were > 19 years of age (M_age_ = 20.1 years, range 16–24 years), lived alone and were mostly male (89%). The average time since they left Syria was 5.3 years, and the mean residence time in Norway was 2 years. Most had Arabic as their mother tongue (76.3%).

**Table 1:** Socio-demographics of the participants

Descriptives	Mean (SD)	range	N (%)
Gender *Female* *Male*			60 (37.5) 100 (62.5)
Age	18.1 (2.4)	13–24	
Years as refugee	5.3 (1.9)	0–10	
Years of residence in Norway	2.0 (1.2)	0–8	
No. of moves	3.7 (2.9)	1–15	
Mother tongue *Arabic* *Kurdish* *Other*			121 (76.3) 34 (21.9) 3 (1.8)
Living with parents *Yes* *No* *Unknown*			120 (75) 35 (21.9) 5 (3.1)

### Descriptives and correlations between study variables

The means and correlation coefficients are presented in Table 2. The participants (n = 151) reported a mean of 4.5 PTEs (SD = 2.64), the most prevalent being *witnessing war* (68%), *feeling your life was in danger* (59%) and *seeing someone die* (55%). A total of 88% reported at least one event, and 61% four or more events. Youth older than 18 years had experienced significantly more PTEs (19–24 years: *t*(151) = -3.60, *p* < 0.001) and boys more so than girls (Boys: *t*(151) = -3.43, *p* = 0.001). Scores for post-migration stressors were an average of 13.56 (SD = 7.56), and dichotomising scores into experiencing each stressor rarely (never, rarely, sometimes) or often (often or very often) showed that participants (n = 127) reported an average of 2.2 post-migration stressors (*SD* = 2.0) they experienced often. The most common were *missing family* (33%), *economic concerns* (29%), *missing previous life* (28%) and *language problems* (26%). Least common was *feeling unsafe* (8%) and *perceived discrimination* (6%). There were no gender differences, but again the youth over 18 years reported more frequent stressors (t(123) = -2.19, *p* = 0.031). The mean score for general mental distress (HSCL-10) (n = 132) was 1.77 (*SD* = 0.61) and 36% had scores above the recommended threshold (≥ 1.85) considering an indication of mental distress [33]. Almost half the participants (48%) had scores above the recommended threshold for PTSD (≥ 17) [36]*.* The mean score for global HRQoL (44.63, *SD* = 10.0) was slightly lower than norm data (48.51 *SD* = 9.28) and 26% reported low HRQoL [38]. All the main study variables were negatively correlated to the main outcome with moderate-to-high correlation coefficients (Small = 0.1 to 0.3, medium = 0.3 to 0.5, large = 0.5 to 1). The negative correlation between PTEs and HRQoL (r = -0.318, p < 0.001) supports the first hypothesis. No correlations between the independent (PTEs) and the mediator variables (Post-migration stressors, mental distress/PTSD) exceeded the 0.80 multicollinearity threshold suggested by Field [39].

**Table 2:** Correlations between main study variables

Variables	1	2	3	4	5	Mean (SD)	Range
1 Global HRQoL	-					44.63 (10.0)	19–84
2 PTE	− .318***	1				4.50 (2.64)	0–10
3 Mental distress	− .572***	.299***	1			1.77 (.61)	1–4
4 PTSS	− .384***	.576***	.416***	1		17.33 (10.22)	0–38
5 Post-migration stressors	− .435***	.556***	.358***	.518***	1	13.56 (7.56)	0–40
6 Age	− .307***	.416***	.143	.149	.323***	18.06 (2.38)	13–24
7 Gender	.001	.273***	− .165	− .011	.110		
8 Residence time	− .104	.245**	.047	.064	.164	2.03 (1.20)	0–8

Global HRQoL: Global Health-related quality of life (KIDSCREEN-10). PTE: Potentially traumatic events pre-migration (Refugee Trauma History Checklist). Mental distress (HSCL-10). PTSS: Post-traumatic stress symptoms (CRIES-8). Post-migration stress (Post-Migration Stress Scale). *p < 0.05. **p < 0.01. ***p < 0.001

### Serial multiple mediation analyses

**Fig. 2:**
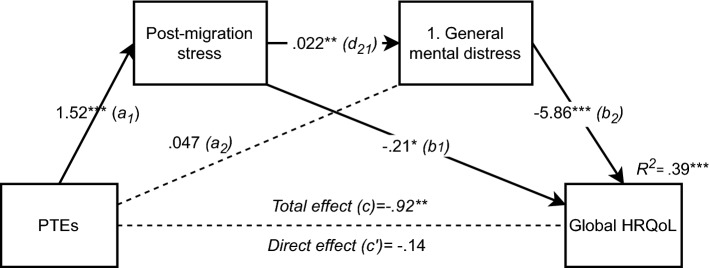
Serial multiple mediation of PTEs’ (Refugee trauma history checklist) relationship to Global HRQoL (KIDSCREEN-10), including post-migration stressors (Refugee post-migration stress scale) as the first mediator and general mental distress (HSCL-10) as a second mediator (n = 117). *p < 0.05. **p < 0.01. ***p < 0.001. Values shown are unstandardised coefficients

The first analysis with post-migration stressors and general mental distress as mediators is presented in Fig. 2.

Supporting our second hypothesis of post-migration stressors as an independent mediator, we found that PTEs influence quality of life indirectly through post-migration stressors and that this indirect effect was significant (*indirect effect (a*_*1*_ and *b*_*1*_*)*: -0.32, 95% CI (-0.66, -0.03)). Hypothesis three was not supported, as general mental distress did not act as an independent mediator (*indirect effect (a*_*2*_ and *b*_*2*_*)*: -0.27, 95% CI (-0.94, 0.67)). However, as the relationship between the mediators (path *d*_*21*_) was positive and significant, there was support for a serial mediation model (hypothesis 4) (*indirect effect (a*_*1*_* d*_*21*_* b*_*2*_*)*: -0.19, 95% CI (-0.42, -0.05)). The model including these variables accounted for 39% of the variance in HRQoL scores (*F*(5,111) = 13.97, *p* < 0.001, *R*^*2*^ = 0.386).

**Fig. 3:**
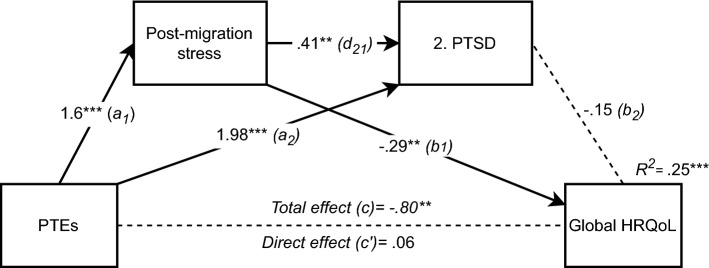
Serial multiple mediation of PTEs’ (refugee trauma history checklist) relationship to Global HRQoL (KIDSCREEN-10), including post-migration stressors (Refugee post-migration stress scale) as the first mediator and PTSD (CRIES-8) as a second mediator (n = 114), *p < 0.05. **p < 0.01. ***p < 0.001. Values shown are unstandardised coefficients

**Fig. 4:**
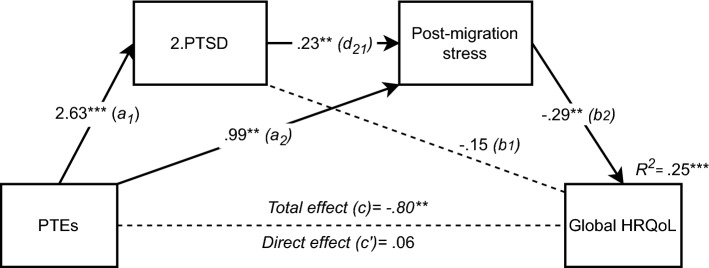
Reversed serial multiple mediation of PTEs’ (refugee trauma history checklist) relationship to Global HRQoL (KIDSCREEN-10), including PTSD (CRIES-8) as the first mediator and post-migration stressors (Refugee post-migration stress scale) as a second mediator (n = 114), *p < 0.05. **p < 0.01. ***p < 0.001. Values shown are unstandardised coefficients

Repeating the same analysis, but replacing general mental distress with PTSD, reiterated the support for the second hypothesis of post-migration stressors as an independent mediator *(indirect effect (a*_*1*_ and *b*_*1*_*)*: -0.46, 95% CI (-0.89, -0.14)), see Fig. 3. Contrary to general mental distress, PTEs were significantly associated with PTSD, even after controlling for post-migration stressors (path *a*_*2*_). There was also a significant association between the two mediators. In fact, PTEs and post-migration stressors contributed to 41% of the variance in PTSD (*F*(5,109) = 19.15, *p* < 0.001, *R*^*2*^ = 0.412). However, when controlling for PTEs, there was no significant association between PTSD and quality of life (path *b*_*2*_), and hypotheses 3 and 4 were therefore not supported in this model. Considering that the direction of influence is unknown, we reversed the order of the mediators in a new analysis (see Fig. 4). This reversal resulted in the confirmation of hypothesis 4 also for PTSD with a small, but significant indirect effect via serial mediation (*indirect effect (a*_*1*_* d*_*21*_* b*_*2*_*)*: -0.18, 95% CI (-0.35, -0.05)). In this reversed model, PTEs are associated with increased PTSD, which in turn increases the frequency of experienced post-migration stressors, which negatively affects quality of life. The total model accounted for 25% of the variance in HRQoL scores (*F*(5,108) = 7.27, *p* < 0.001, *R*^*2*^ = 0.251).

### Types of post-migration stressors associated with different dimensions of HRQoL

Lastly, we explored the pathway between post-migration stressors and HRQoL further by analysing types of stressors and dimensions of HRQoL as well as the global HRQoL used in the mediation models (see Table 3).

**Table 3:** Correlations between post-migration stressors (organised by descending mean item scores) and dimensions of HRQoL (KIDSCREEN-27) and Global HRQoL (KIDSCREEN-10)

	Mean (SD)	Global HRQoL	Physical Wellbeing	Psychological wellbeing	Parents/ Autonomy	Friends	School
I worry about not having enough money	1.78 (1.30)	− .436***	− .164	− .484***	− .360***	− .278**	− .334***
I find schoolwork difficult because of language problems	1.77 (1.22)	− .246**	− .202*	− .244**	− .209*	− .190*	− .241**
I miss things I used to do before I came to Norway	1.70 (1.36)	− .189*	− .098	− .211*	− .102	− .049	− .134
I am sad because I miss my family	1.70 (1.66)	− .211*	− .050	− .232*	− .063	− .150	− .146
I worry about having to move again	1.53 (1.29)	− .301***	− .066	− .308***	− .104	− .154	− .175
I feel lonely or like an outsider	1.31 (1.27)	− .277**	− .149	− .229*	− .164	− .157	− .196*
I feel split between my parents and my friends’ expectations about how to behave	1.15 (1.27)	− .271**	− .012	− .301***	− .245**	− .165	− .129
I worry about not being able to stay in Norway	1.20 (1.26)	− .210*	− .059	− .148	− .251**	− .108	− .128
I felt badly treated because of my background	.81 (1.13)	− .313***	− .272**	− .226*	− .246**	− .142	− .224*
I feel unsafe in my neighbourhood	.61 (1.05)	− .149	− .033	− .119	− .114	− .153	− .085
Total post-migration stressors	13.56 (7.56)	− .457***	− .208*	− .434***	− .320***	− .243**	− .295***

Spearman’s Rho is used due to skewness and ordinal data. *p < 0.05. **p < 0.01. ***p < 0.001

Significance after Bonferroni–Holmes correction is shown in bold

*Economic concerns* had the highest means and showed significant correlation with almost all dimensions. The second last item, *perceived discrimination*, also had significant correlation across several outcomes despite having lower means. Two of the items with higher means (*missing family* and *my previous life*) did not affect the outcomes greatly. Global HRQoL and *psychological wellbeing* were affected by several of the same stressors, while few stressors were significant for *physical wellbeing*. *Worrying about being able to stay in Norway* was uniquely associated with the dimension for Parents/Autonomy.

## Discussion

### Predictors, mediators and outcome levels

The aim of this study was to explore how experiences from war and forced migration influence quality of life after recent resettlement. Based on the “Ecological model of refugee distress”, we explored models including both post-migrations stressors and mental distress as mediators. We found that PTEs reduced quality of life and that this was mediated by post-migration stressors alone or in sequence with mental distress, but not by mental distress alone. The participants had high levels of reported PTEs (61%), general mental distress (36%) and PTSD (48%), akin to other studies of resettled Syrian children and adolescents [4, 5, 40–42]. The HRQoL was moderately good but lower than population norms [38]. The level of post-migration stressors was slightly lower compared to Syrian adults [43] or other resettlement contexts such as refugee camps [16], low-income host countries [15] and among asylum seekers [17, 44].

### Serial mediation analyses

Our analyses suggest that negative experiences from forced migration reduce quality of life after resettlement, as proposed in our first hypothesis. As proposed in the “Ecological model of refugee distress”, post-migration stressors independently and fully mediated the relationship between PTEs and HRQoL, supporting its relevance and as a mediator also for HRQoL. This reflects earlier findings of post-migration stressors as a mediator to mental distress [7, 10, 15–18] but is contrary to the results of a recent meta-analysis that found no mediating effect of post-migration stressors on wellbeing indicators [7]. This could be due to only a handful of studies being relevant and the use of different measures in a variety of contexts, but is also an indication of the need to replicate our results in larger studies. Although numerous studies have found that mental distress predict quality of life [27, 28], few have investigated this as a mediator between trauma and quality of life as we proposed in our third hypothesis. One study of adult Ethiopians in refugee camps found that trauma from displacement had both a direct effect upon HRQoL and an indirect effect through mental distress [29]. However, in our study mental distress did not act as an independent mediator, and hypothesis three was not supported. The different results could be due to differences in age, contexts and levels of distress. Conversely, we found that mental distress acted as a mediator in conjunction with post-migration stressors, supporting our fourth hypothesis, and reaffirming the importance of including both factors when assessing HRQoL in refugees.

Longitudinal studies on refugee youth suggest differential impact of pre- and post-migration factors throughout the resettlement process, as traumatic experiences before arrival predict short-term reactions and stressors in exile better predict psychological problems a decade after arrival [45]*.* In the present study, the impact of pre- and post-factors also varied with type of mental distress, as post-migration stressors seemed to be more relevant for general mental distress and explained more variance in HRQoL, whilst PTEs seemed to have more relevance for PTSD and explained less of the variance. These differences have also been found in other studies [46] and suggest that discussions on pre- and post-migration influence should not be an either/or debate, but relate to their differential impact throughout the settlement process. Type of mental distress is also pertinent to consider in addition to levels above thresholds, as there was no significant association between PTSD and HRQoL after controlling for earlier experiences (PTEs), despite almost half of the participants scoring above the thresholds.

A cumulative effect of post-migration stressors was supported by our analyses, but further exploration also revealed differences between types of stressors and dimensions of HRQoL. Global HRQoL and the dimension of psychological wellbeing were most affected by post-migration stressors and physical wellbeing the least, suggesting that other factors (e.g. somatic symptoms) could be relevant for the latter. Some stressors were relevant across several dimensions, notably *economic concerns* and *perceived discrimination*, which are repeatedly found as detrimental to health and wellbeing [3, 7, 47]. In contrast, two of the most commonly reported experiences (*missing family or previous life*) had low correlation, possibly indicating less severe emotional distress [17]. These results reflect the “ecological model of refugee distress” where factors at multiple levels influence health and wellbeing. They also inspire hope as the most influential stressors are malleable factors. However, it also highlights the responsibility of host nations for protecting against or alleviating unnecessary pressure on an already vulnerable population as the most influential types of stressors seem to be embedded in social structures.

As mentioned earlier**,** Miller and Rasmussen [12] suggested that post-migration stressors could deplete coping mechanisms, affecting the capacity to recover from trauma. Other theories, such as the **“**Stress sensitivity theory” propose that past trauma activates an overreaction to ongoing demands thus decreasing the tolerance for stressors perceived as manageable by others [48]. These could be explanations as to why increasing numbers of PTEs were associated with higher frequencies of post-migration stressors in our study. Emotion dysregulation has been found to mediate mental distress in refugees [4, 49]. Also, psychological processes such as emotion regulation, memory and executive functions are susceptible to the influence of trauma or elevated stress during development with long-lasting effects, as this in turn affects the capacity to regulate future stress responses [50]. The proposed model might also be reversed in a “Stress generation model” [51], as tested in Fig. 4. Mental distress would then make people behave and react in ways that create more stressful situations, for example, by avoiding social interaction or making impulsive financial choices. The general burden of mental distress could also act as a worry or stressor in itself and is in fact included in some post-migration stressor scales [52]. The interaction between distress and stressors suggests a bidirectional relationship where both processes occur, also shown in other studies [53, 54], creating a more complex and transactional model [51]. These proposed processes could inform current treatments for mental distress or general psychosocial interventions in schools. For example, the models could help explain why some experience reduced symptom burden after treatment for PTSD but not increased quality of life, and vice versa. Implications for a therapeutic approach would be the importance of addressing currents stressors, not only symptoms, as failure to do so may limit the effectiveness of treatment [10] and also to include holistic treatment goals such as quality of life. Lastly, broader and structural interventions, including all refugee youth, may alleviate or prevent post-migration stressors and mental distress and should be implemented by host nations.

Although psychological problems are frequent in refugee children, the extents are reduced over time in settlement [13, 45] and wellbeing remains high [23]. This could be a sign of natural recovery processes or resilience, where youth develop new resources such as cultural competence, language skills or networks. Resettlement can therefore be stressful, but also involve personal growth and resilience [55], and as such it supports Antonovsky’s criticism of early stress theories for assuming that stressors were inherently negative and the importance of focussing on health resources [56]. Social support and positive coping styles are suggested as protective factors buffering mental distress in refugee children and youth [3, 4] and are also associated with increased quality of life [18, 27]. Our models did not investigate any protective factors, and further studies should include such measures.

## Limitations

Limitations of this study relate to its cross-sectional nature and the interlinked nature of variables under analysis, which prevents any assertion of causality and direction. Although the dose–response association between the number of PTEs and mental distress is well documented in the literature, an additional limitation of this analysis was the equal weighting of such events. Similar events might incur different reactions in individuals and, depending upon the type of event, one event might be enough to qualify as the highest trauma. Also, the measure for post-migration stressors was not validated for this sample and therefore some items might not be appropriate for the group. Despite evidence of resilience factors’ significance, these models did not include protective factors which future studies should include. A non-probabilistic sample of recently resettled youth with few participants does not reflect the true prevalence of mental distress, although the levels were similar to other studies. Even though the sample used in our data was comparable to Syrians registered in Norway at the time, the generalisability of our findings to other countries or other groups of immigrant youth remains a question to be investigated in further research.

## Conclusion

Building on previous knowledge, our models propose that experiences from forced migration influence quality of life negatively through increasing mental distress and post-migration stressors. Despite high levels of mental distress, these symptoms did not affect HRQoL directly, but only through an increase in post-migration stressors. There were differences between the two types of mental distress analysed and their relationship between the variables. Post-migration stressors mediated the influence of PTEs on HRQoL, independently and in serial mediation with mental distress. This reiterates the importance of addressing current stressors, which must be a focus of host nation policies. This would also reframe attention from individual capabilities often targeted in interventions to more structural factors in the resettlement environments, as the most influential types of post-migration stressors seem to be embedded in social structures. HRQoL is a relevant measure for refugee youth in public health surveillance and as an outcome of interventions, and this study adds to the knowledge of psychological processes important for wellbeing in refugee youth. However, future research should include protective and resilience factors to further our understanding on how to promote positive development and quality of life in refugee youth after resettlement.

## Data Availability

Not applicable.

## Footnotes

1 * Terms related to the period before, during and after flight or forced migration are used interchangeably in research. In this article, the terms pre- and post-migration are chosen to reflect the measures used. Pre-migration includes experiences or events prior to and during flight (Potentially Traumatic Events), whilst post-migration refers to factors after arrival or resettlement in a host country, independent of asylum status.*
